# Globular Adiponectin Inhibits Lipopolysaccharide-Primed Inflammasomes Activation in Macrophages via Autophagy Induction: The Critical Role of AMPK Signaling

**DOI:** 10.3390/ijms18061275

**Published:** 2017-06-15

**Authors:** Mi Jin Kim, Eun Hye Kim, Nirmala TiliJa Pun, Jae-Hoon Chang, Jung-Ae Kim, Jee-Heon Jeong, Dong Young Choi, Sang-Hyun Kim, Pil-Hoon Park

**Affiliations:** 1 College of Pharmacy, Yeungnam University, Gyeongsan 712-749, Korea; darkylove@naver.com (M.J.K.); eun_h1209@naver.com (E.H.K.); tilijan@hotmail.com (N.T.P.); jchang@yu.ac.kr (J.-H.C.); jakim@yu.ac.kr (J.-A.K.); jeeheon@yu.ac.kr (J.-H.J.); dychoi@yu.ac.kr (D.Y.C.); 2 Department of Pharmacology, School of Medicine, Kyungpook National University, Daegu 700-422, Korea; shkim72@knu.ac.kr

**Keywords:** adiponectin, 5′AMP-activated protein kinase, autophagy, caspase, inflammasomes, interleukins

## Abstract

The inflammasome acts as a key platform for the activation of pro-inflammatory cytokines. Adiponectin exhibits potent anti-inflammatory properties. However, the effect of adiponectin on the modulation of the inflammasome has not been explored. Herein, we show that globular adiponectin (gAcrp) suppressed lipopolysaccharide (LPS)-primed inflammasomes activation in murine peritoneal macrophages judged by prevention of interleukin-1β (IL-1β) maturation, caspase-1 activation, apoptosis-associated speck-like protein containing a caspase recruitment domain (ASC) speck formation, and pyroptotic cell death. Interestingly, pretreatment with 3-methyl adenine, a pharmacological inhibitor of autophagy, abrogated the suppressive effects of gAcrp on IL-1β secretion and caspase-1 activation, indicating the crucial role of autophagy induction in gAcrp-modulation of the inflammasome activation. In addition, inhibition of 5′Adenosine monophaspahate (AMP)-activated protein kinase (AMPK) signaling abolished suppressive effect of gAcrp on inflammasomes activation. Furthermore, autophagy induction or inhibition of the inflammasome activation by gAcrp was not observed in macrophages deficient in AMPK. Taken together, these results indicate that adiponectin inhibits LPS-primed inflammasomes activation in macrophages via autophagy induction and AMPK signaling-dependent mechanisms.

## 1. Introduction

The inflammasome, a multimeric cytosolic protein complex comprising (pro)-caspase-1, nucleotide-binding oligomerization domain-like receptor (NLR) protein, and apoptosis-associated speck-like protein containing a caspase recruitment domain (ASC), is activated upon recognition of various danger signals, including exogenous pathogen-associated molecular patterns (PAMPs) and endogenous danger-associated molecular patterns (DAMPs) [1]. The inflammasome functions as signaling platforms for the activation of pro-inflammatory cytokines, particularly interleukin family cytokines, via caspase-1 [2,3]. Activation of the inflammasome further induces pyroptosis, a pro-inflammatory type of cell death distinct from apoptosis [4]. The maturation and secretion of interleukin family cytokines and pyroptosis are considered critical mediators of tissue damage and metabolic imbalances. Emerging evidence indicates a potential role for the inflammasome activation in the pathogenesis of a number of diseases associated with inflammation. For example, inhibition of the inflammasome directly led to the suppression of inflammation in adipose tissues and improved insulin-sensitivity in obese patients with type 2 diabetes [5], and blockade of interleukin-1β (IL-1β) resulted in a reduction in gout symptoms [6], suggesting that modulation of inflammasomes could be a promising strategy for the treatment of various inflammatory diseases.

Activation of the inflammasome is initiated by stimulation of pattern recognition receptors (PRRs), which leads to the assembly of inflammasomes components and causes maturation of interleukins in a series of consecutive processes. Typically, the NLR protein is associated with the adaptor protein, namely ASC, which recruits pro-caspase-1 and leads to the proteolytic activation of caspase-1. Subsequently, activated caspase-1 induces proteolytic maturation and secretion of interleukin family cytokines in immune cells [2,7]. Of the various types of NLR proteins, *NACHT*, *LRR* and *PYD* domains-containing protein 3 (NLRP3) are the most common in macrophages and the best characterized to date [8,9].

Autophagy is a conserved intracellular self-digestive process that involves the delivery of autophagosome-lysosome machinery to damaged organelles or proteins and removal of target components for the maintenance of cellular homeostasis [10]. Each step in the autophagic process is coordinately regulated by a number of genes associated with autophagy, including *ATG5* (autophagy related 5), *BECN-1* (Beclin-1), and *LC3* (microtubule-associated protein 1 light chain 3) [10,11]. Although the autophagic process occurs even in normal conditions, it is commonly promoted in physiologically stressful conditions such as starvation, oxidative stress, and accumulated misfolded proteins [12]. There is a growing appreciation that the induction of autophagy plays important roles in various biological responses and that defects in autophagy are directly linked to many human diseases, including cancer, neurodegenerative diseases, and aging [13]. Recent studies have indicated that autophagy also plays a critical role in the modulation of innate immunity and inflammation. For example, genetic ablation of autophagy enhanced production of IL-18 and IL-1β mediated by Toll-like receptor-4 (TLR-4) [14], whereas induction of autophagy negatively regulated IL-1β production [15]. In addition, depletion of autophagy-related gene products has been shown to enhance accumulation of dysfunctional mitochondrial DNA (mtDNA), resulting in the secretion of inflammatory cytokines [16]. These reports collectively suggest the prominent role of autophagy in the modulation of inflammatory conditions.

Adiponectin, an adipokine produced primarily in adipose tissue, was originally reported to play a critical role in lipid metabolism and insulin sensitization [17]. In addition to its well-known metabolic effects, adiponectin exhibited potent anti-inflammatory properties mediated via multiple mechanisms, including inhibition of NF-κB signaling and inflammatory cytokine production, and/or induction of anti-inflammatory genes such as heme oxygenase-1 [18,19,20]. Once secreted, part of adiponectin (called as full length adiponectin) is subjected to proteolytic cleavage at sites in the collagenous domain. As a result, the fragment of carboxyl-terminal containing globular domain of adiponectin is produced. Although the cleaved globular adiponectin is a minor isoform in the plasma, the globular adiponectin has been shown to possess more potent effect on fatty acid oxidation (and weight loss) and anti-inflammatory effects than full length adiponectin [21,22]. Accumulating evidence has indicated that the anti-inflammatory effects by globular adiponectin can be mediated by the induction of autophagy. For instance, adiponectin was shown to diminish angiotensin II-induced inflammatory responses via 5′AMP-activated protein kinase (AMPK)-mediated autophagy activation [23]. In addition, induction of autophagy contributes to the development of tolerance to LPS-induced (LPS-induced) tumor necrosis factor-α (TNF-α) expression by globular adiponectin in macrophage [19]. Based on the previous reports, it is well established that adiponectin acts as a potent regulator of inflammatory responses. However, the modulatory roles of adiponectin in inflammasomes activation and its role in the regulation of inflammation are not well defined.

AMPK, a sensor molecule involved in the maintenance of cellular energy homeostasis, is regarded as one of the key mediators of the beneficial effects of adiponectin [24]. In addition to its critical role in metabolic homeostasis, recent studies have implicated AMPK signaling in autophagy induction [25]. Knockdown of AMPK prevented resveratrol-stimulated autophagy in leukemia cells [26] and depletion of AMPK limited adiponectin-induced autophagy activation, which contributes to prevent ethanol-induced apoptosis in HepG2 cells [27]. Furthermore, inactivation of AMPK by palmitate resulted in the suppression of *LC3II* expression and formation of auto-phagosomes in bone marrow-derived macrophages [28], indicating the crucial role of AMPK signaling in autophagy induction.

Inflammasomes activation, leading to the maturation of inflammatory cytokines and pyroptosis of immune cells, acts as an important signaling platform in the development of inflammatory diseases. Although adiponectin is well known to possess potent anti-inflammatory properties, the modulatory effect of adiponectin on the inflammasome has not been explored. Thus, to better understand the mechanisms underlying the anti-inflammatory action of adiponectin, we investigated the effects of globular adiponectin on LPS-primed inflammasomes activation and IL-1β production and its potential mechanisms underlying these effects. Herein, we demonstrated for the first time that globular adiponectin suppresses LPS-stimulated IL-1β production via modulation of the inflammasome in macrophages. Furthermore, autophagy induction and AMPK signaling play pivotal roles in the suppression of inflammasomes activation by globular adiponectin.

## 2. Results

### 2.1. Globular Adiponectin Inhibits LPS-Stimulated IL-1β Maturation and Secretion by Murine Peritoneal Macrophages

**Figure 1:**
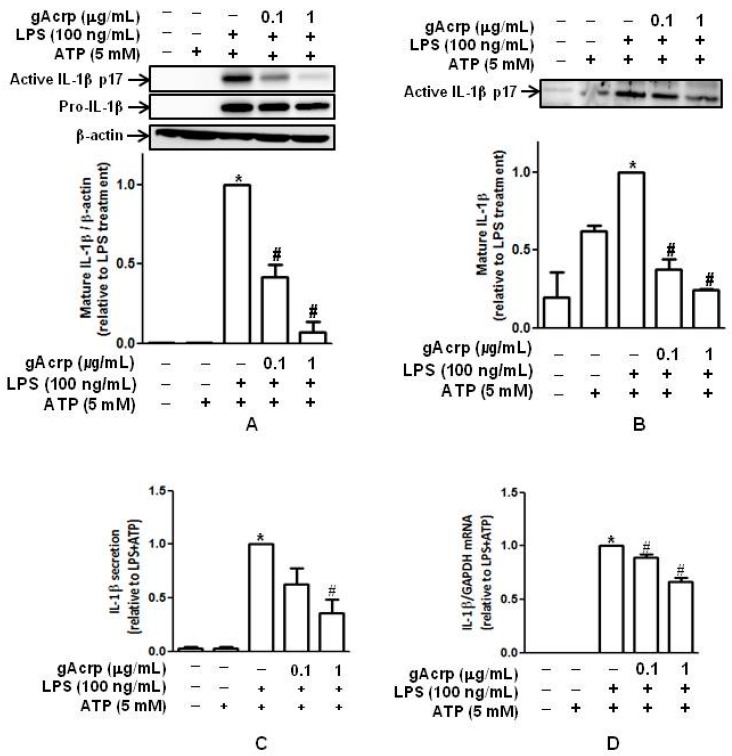
Effects of globular adiponectin on maturation and secretion of IL-1β stimulated by lipopolysaccharide (LPS) in murine peritoneal macrophages. (**A**) Macrophages were isolated from murine peritonea, pretreated with the indicated concentrations of gAcrp for 18 h, and stimulated with LPS (100 ng/mL) for 8 h and then ATP (5 mM) for 1 h. Total cellular lysates were prepared, and the levels of pro- and mature active IL-1β were determined by Western blot analysis. Representative images from three independent experiments are shown along with β-actin as an internal loading control; (**B**) after treatment with gAcrp, LPS, and ATP, as in Figure 1A, media were collected, and the levels of mature active IL-1β were measured by Western blot analysis. Quantitative analysis of active IL-1β expression was performed by densitometric analysis and shown in the lower panel (**A**,**B**). Values presented are fold change compared to the cells treated with LPS and ATP and expressed as mean ± SEM (*n* = 3 for **A**, *n* = 2 for **B**). * *p* < 0.05 compared to the control cells. # *p* < 0.05 compared to the cells treated with LPS and ATP; (**C**) primary macrophages were isolated from murine peritonea, treated with gAcrp for 18 h, and stimulated with LPS (100 ng/mL) for 24 h and ATP for 1 h. The amount of secreted IL-1β was determined by ELISA as described in materials and methods. Results are presented as the mean ± SEM, *n* = 3. * *p* < 0.05 compared to control cells; # *p* < 0.05 compared to cells treated with LPS and ATP; (**D**) peritoneal macrophages were pretreated with gAcrp for 18 h, followed by stimulation with LPS (100 ng/mL) for 8 h and ATP for 1 h. IL-1β mRNA levels were determined by quantitative RT-PCR as indicated in materials and methods and normalized to the levels of GAPDH mRNA. Values indicate the fold increase compared to cells treated with LPS plus ATP and are expressed as the mean ± SEM (*n* = 4). * *p* < 0.05 compared with untreated cells; # *p* < 0.05 compared to cells treated with LPS and ATP.

Activation of the inflammasome leads to the generation of active pro-inflammatory cytokines, including IL-1β. To investigate the possible regulatory effect of adiponectin on inflammasomes, we first examined whether globular adiponectin (gAcrp) inhibits maturation and secretion of IL-1β by macrophages. In these experiments, macrophages were isolated from murine peritonea and pretreated with gAcrp, followed by priming with LPS and further stimulation with ATP. As shown in Figure 1A, LPS stimulated maturation of IL-1β in the presence of ATP in murine peritoneal macrophages, consistent with previous reports, whereas pretreatment with gAcrp almost completely inhibited LPS-induced IL-1β maturation. To confirm the suppressive effect of gAcrp on IL-1β maturation, we collected culture media and measured the levels of active IL-1β secreted. As indicated in Figure 1B, pretreatment with gAcrp also significantly suppressed IL-1β maturation in a dose-dependent manner, similar to the results observed from cellular lysates. Pretreatment with gAcrp elicited similar regulatory effects on IL-1β secretion, as determined by ELISA (Figure 1C). In these experiments, LPS treatment, as a first signal, significantly increased pro-IL-1β protein expression, which was substantially inhibited by pretreatment with gAcrp (middle panel in Figure 1A). However, the suppressive effect on pro-IL-1β protein expression was much less than the inhibition of IL-1β maturation (comparing the expression levels of pro-IL-1β and mature IL-1β in Figure 1A). Globular adiponectin also significantly inhibited LPS-stimulated mRNA expression of IL-1β (Figure 1D). However, the suppressive effect by gAcrp was also lower than the regulation of IL-1β maturation, which was similar to the regulation of pro-IL-1β expression. Collectively, these results indicate that adiponectin effectively prevents LPS-induced maturation and secretion of IL-1β, as well as pro-IL-1β expression, in macrophages.

### 2.2. Globular Adiponectin Suppresses LPS-Primed Activation of Caspase in Peritoneal Macrophages

**Figure 2:**
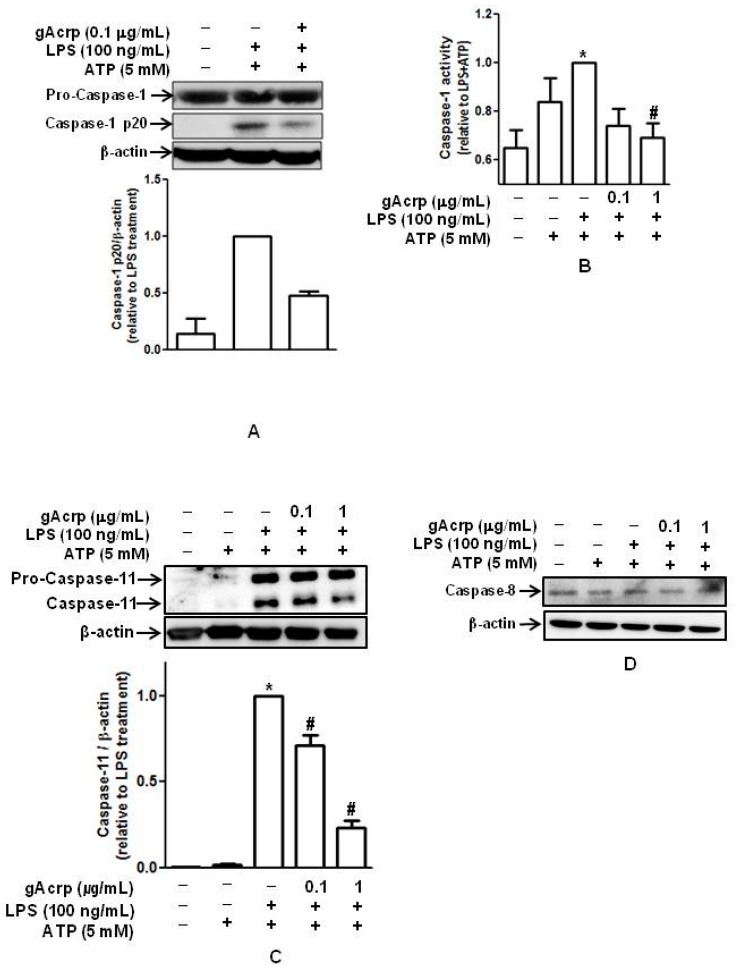
Effects of globular adiponectin on lipopolysaccharide (LPS)-primed caspase-1 activation in peritoneal macrophages**.** Peritoneal macrophages were pretreated with gAcrp (0.1 μg/mL) for 18 h, followed by stimulation with LPS (100 ng/mL) for 6 h and then treatment with ATP (5 mM) for 30 min. (**A**) Pro- and cleaved active forms of caspase-1 were detected by Western blot analysis in peritoneal macrophages. Representative images from three independent experiments are shown along with β-actin as an internal loading control. Quantitative analysis of caspase-1 p20 expression was performed by densitometric analysis and shown in the lower panel. Values presented are fold change compared to LPS and ATP treatment and represented as mean ± SEM (*n* = 3). * *p* < 0.05 compared to the control cells. # *p* < 0.05 compared to the cells treated with LPS and ATP; (**B**) cell lysates were prepared and used for the measurement of caspase-1 enzyme activity. Data are expressed as fold changes relative to cells treated with LPS and ATP. Values are presented as the mean ± SEM, *n* = 4. * *p* < 0.05 compared to cells treated with ATP; # *p* < 0.05 compared to cells treated with LPS and ATP; (**C**) Caspase-11 expression was measured by Western blot analysis. Representative images are shown along with β-actin as an internal loading control. Quantitative analysis of caspase-11 expression was performed by densitometric analysis and shown in the lower panel. Values presented are fold change compared to LPS and ATP treatment and presented as mean ± SEM (*n* = 2). * *p* < 0.05 compared to the control cells. # *p* < 0.05 compared to the cells treated with LPS and ATP; (**D**) Caspase-8 expression levels were determined by Western blot analysis. Representative images from three independent experiments are shown along with β-actin as an internal loading control.

We next assessed the effect of gAcrp on the activity of caspase-1, which plays a key role in processing of pro-inflammatory cytokines and is regarded as a critical indicator of the inflammasome activation [29]. As shown in Figure 2, treatment with gAcrp substantially suppressed the generation of cleaved active caspase-1 in primary peritoneal macrophages (Figure 2A) without significant effect on the expression of pro-caspase-1. Similarly, caspase-1 enzymatic activity primed by LPS was significantly diminished in the presence of gAcrp (Figure 2B). In addition to caspase-1, recent studies have shown that different types of caspases, including caspase-8 and caspase-11, are able to induce maturation of pro-IL-1β in macrophages [30,31]. Thus, we examined the modulatory effects of gAcrp on caspase-8 and caspase-11. Treatment with gAcrp significantly suppressed LPS-induced caspase-11 expression (Figure 2C), suggesting that caspase-11 may be involved in modulation of IL-1β by gAcrp. However, in the modulation of caspase-8, LPS treatment did not significantly increase caspase-8 expression, and gAcrp did not significantly affect caspase-8 expression in our experimental conditions (Figure 1D). Furthermore, LPS and gAcrp treatments did not significantly modulate enzymatic activity of caspase-8 (Figure S1), confirming that caspase-8 does not play a role in the modulation of IL-1β by gAcrp.

### 2.3. Globular Adiponectin Suppresses Inflammasomes Activation and Pyroptosis in LPS-Primed Peritoneal Macrophages

**Figure 3:**
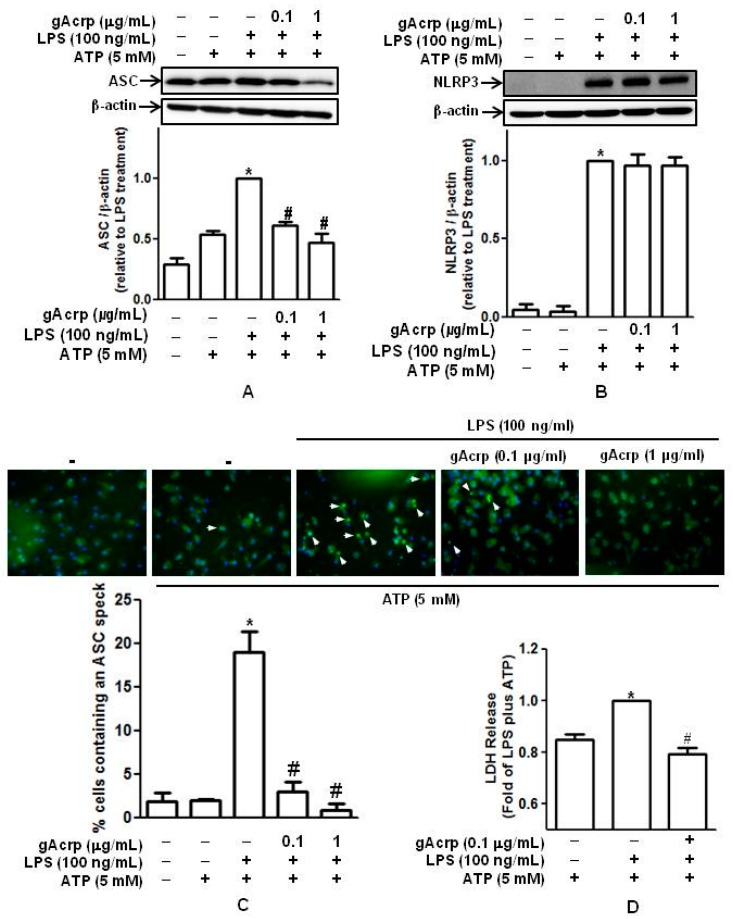
Effects of globular adiponectin on the inflammasome activation and lactate dehydrogenase (LDH) release in murine peritoneal macrophages treated with lipopolysaccharide (LPS). Peritoneal macrophages were pretreated with gAcrp (0.1 μg/mL) for 18 h, stimulated with LPS (100 ng/mL) for 6 h, and then treated with ATP (5 mM) for 1 h. (**A**) ASC expression levels were determined by Western blot analysis. Representative images from three independent experiments are shown along with β-actin as an internal loading control; (**B**) *NLRP3* expression levels were determined by Western blot analysis. Representative images from three independent experiments are shown along with β-actin as an internal loading control. Quantitative analyses for the measurement of ASC (**A**) and NLRP3 (**B**) expression were performed by densitometric analysis and shown in the lower panel. Values presented are fold change compared to LPS and ATP treatment and expressed as mean ± SEM (*n* = 3). * *p* < 0.05 compared to the control cells. # *p* < 0.05 compared to the cells treated with LPS and ATP; (**C**) cells were stained with a specific antibody against ASC (green) and DAPI (blue). ASC speck formation was analyzed by fluorescent immunocytochemistry and is indicated by white arrows. Representative images from three independent experiments are presented. Quantitative analysis of ASC speck formation (dots) was performed by counting the cells that showing ASC-DAPI and shown in the lower panel. Values are expressed as a percentage of the cells that showing ASC-DAPI dots. * *p* < 0.05 compared to cells treated with ATP; # *p* < 0.05 compared to cells treated with LPS and ATP; (**D**) cell culture media were collected and used to measure LDH release. Values are shown as the mean ± SEM, *n* = 3. * *p* < 0.05 compared with cells treated with ATP; # *p* < 0.05 compared with cells treated with LPS and ATP.

ASC and NLRP3, in addition to caspase-1, are critical components of the inflammasome. To further elucidate the impact of gAcrp on the inflammasome, we examined the effects of gAcrp on the expression of ASC and NLRP3 and observed that LPS-stimulated ASC expression was dose-dependently decreased by gAcrp (Figure 3A), whereas NLRP3 induction was not significantly affected by gAcrp (Figure 3B). In addition to the expression of ASC and NLRP3, ASC speck formation (oligomerization) is regarded as a marker of the inflammasome assembly [7]. We therefore next analyzed the effects of gAcrp on ASC speck formation by fluorescent microscopy. As shown in Figure 3C, treatment of peritoneal macrophages with LPS and ATP stimulated ASC speck formation. However, ASC speck formation was significantly reduced by pretreatment with gAcrp, indicating the suppression of inflammasomes assembly.

**P**yroptosis, a type of programmed cell death distinct from apoptosis, is characterized by the loss of plasma membrane integrity and release of intracellular contents into the extracellular milieu following inflammasomes activation [32,33,34]. We next examined the regulatory effect of gAcrp on pyroptosis by LDH assay. LPS and ATP treatment elicited a significant increase in the LDH released from murine peritoneal macrophages, whereas pretreatment with gAcrp substantially inhibited LDH release (Figure 3D), implying the protective effect of gAcrp from pyroptotic cell death.

### 2.4. Autophagy Induction Plays A Critical Role in The Suppression of IL-1β Production by Globular Adiponectin in Murine Peritoneal Macrophages

**Figure 4:**
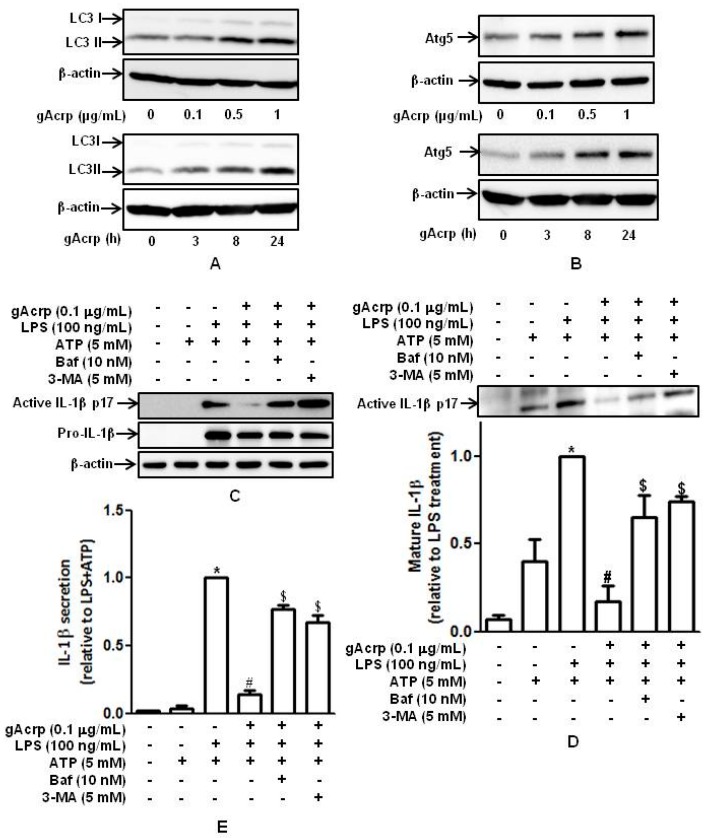
Role of autophagy induction in the suppression of IL-1β maturation and secretion by globular adiponectin in macrophages. (**A**,**B**) Macrophages were treated with gAcrp (0.1 μg/mL) for 18 h. *ATG5* (**A**) and *LC3* (**B**) expression levels were determined by Western blot analysis. Representative images from three independent experiments are shown along with β-actin as an internal loading control. (**C**–**E**) Cells were pretreated with gAcrp (0.1 μg/mL) for 18 h in the absence or presence of 3-MA or Bafilomycin, stimulated with LPS (100 ng/mL) for 8 h, and then treated with ATP (5 mM) for 1 h; (**C**) total cellular lysates were prepared, and the levels of pro- and mature active IL-1β were measured by Western blot analysis. Representative images from three independent experiments are shown along with β-actin as an internal loading control; (**D**) cell culture media were collected, and the levels of mature active IL-1β were measured by Western blot analysis. Quantitative analysis of active IL-1β (p17) was performed by densitometric analysis and shown in the lower panel. Values presented are fold change compared to LPS and ATP treatment and expressed as mean ± SEM (*n* = 3). * *p* < 0.05 compared to the control cells. # *p* < 0.05 compared to the cells treated with LPS and ATP. $ *p* < 0.05 compared to the cells treated with gAcrp and LPS/ATP. (E) The amount of secreted IL-1β was determined by ELISA. Results are presented as the mean ± SEM, *n* = 3. * *p* < 0.05 compared with control cells; # *p* < 0.05 compared with the cells treated with LPS and ATP. $ *p* < 0.05 compared to the cells treated with gAcrp and LPS/ATP.

Autophagy, acting as a modulatory process of innate immune system, is known to regulate inflammatory cytokine production [14,15]. We next investigated whether induction of autophagy plays a role in the modulation of IL-1β maturation and inflammasomes formation by adiponectin. To test this, we first confirmed the autophagy inducing effects of gAcrp in our experimental conditions. As indicated in Figure 4, treatment with gAcrp induced a significant increase in the expression of *ATG5* and *LC3II* in a dose-and time-dependent manner in our experimental conditions (Figure 4A,B), consistent with previous reports. To further investigate the functional role of autophagy induction in the modulation of IL-1β maturation by adiponectin, we assessed the effect of pharmacological autophagy inhibitors on the modulation of IL-1β maturation by gAcrp. Interestingly, both treatment with 3-MA, an inhibitor of type III PI3K, and bafilomycin, an inhibitor of fusion between autophagosomes and lysosomes, substantially abrogated suppression of LPS-stimulated IL-1β maturation by gAcrp (Figure 4C). In addition, secretion of the mature active form of IL-1β was also significantly restored by treatment with Bafilomycin or 3-MA (Figure 4D), which was determined by Western blot analysis using cell culture media. Finally, the levels of IL-1β secreted into media were also restored by 3-MA or Bafilomycin, as assessed by ELISA (Figure 4E). Taken together, these results imply that autophagy induction plays a crucial role in the suppression of IL-1β production by gAcrp. To further examine the role of autophagy induction in modulating the production of other pro-inflammatory cytokines by gAcrp, we investigated whether induction of autophagy also plays a role in the suppression of IL-6 production by gAcrp. We observed that treatment with 3-MA did not significantly affect IL-6 expression (Figure S2), indicating that autophagy is involved in modulation of inflammatory cytokines production by adiponectin in a gene-selective manner.

### 2.5. Autophagy Induction Contributes to The Suppression of Caspase-1 Activation, ASC Speck Formation, and Pyroptosis by Globular Adiponectin

**Figure 5:**
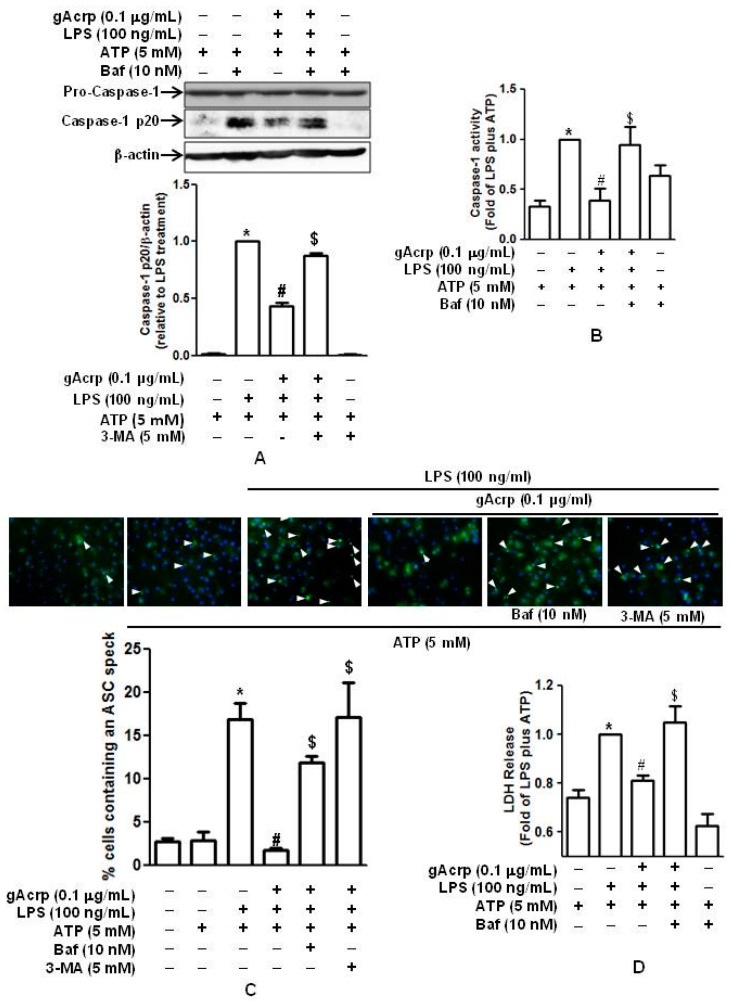
Role of autophagy induction in the suppression of caspase-1 activation, inflammasomes activation, and pyroptosis by globular adiponectin. (**A**–**D**) Cells were pretreated with gAcrp (0.1 μg/mL) for 18 h in the absence or presence of 3-MA (5 mM), stimulated with LPS (100 ng/mL) for 6 h, and then treated with ATP (5 mM) for 30 min. * *p* < 0.05 compared to cells treated with ATP; # *p* < 0.05 compared to cells treated with LPS and ATP; $ *p* < 0.05 compared to cells treated with gAcrp and LPS/ATP. (**A**) Levels of pro- and cleaved forms of caspase-1 in murine peritoneal macrophages were measured by Western blot analysis. Quantitative analysis of cleaved caspase-1 (caspase-1 p20) expression was performed by densitometric analysis and shown in the lower panel. Values presented are fold change compared to LPS and ATP treatment and represented as mean ± SEM (*n* = 3). * *p* < 0.05 compared to the control cells. # *p* < 0.05 compared to the cells treated with LPS and ATP. $ *p* < 0.05 compared to the cells treated with gAcrp and LPS/ATP; (**B**) the culture media from peritoneal macrophages were prepared and used in a caspase-1 enzyme activity assay. Data are expressed as fold changes relative to LPS and ATP treatment. Values are shown as the mean ± SEM, *n* = 5; (**C**) cells were stained with antibody specific for ASC (green) and DAPI (blue). ASC speck formation was analyzed by fluorescent immunocytochemistry. Representative images from three independent experiments are shown along with quantitation of ASC speck formation (dots) in the lower panel. Values are expressed as a percentage of the cells that showing ASC-DAPI dots. * *p* < 0.05 compared to cells treated with ATP; # *p* < 0.05 compared to cells treated with LPS and ATP; (**D**) cell culture media were prepared and used for measurement of the LDH released. Values are presented as the mean ± SEM, *n* = 4.

We next investigated whether autophagy induction is involved in the modulation of caspase-1 activation by gAcrp. As shown in Figure 5A, pretreatment with 3-MA abrogated the suppressive effect of gAcrp on cleavage of pro-caspase-1 in primary murine peritoneal macrophages without significant effect on the expression of pro-caspase-1. In addition, treatment with 3-MA also restored suppression of caspase-1 enzymatic activity by gAcrp (Figure 5B), similar to the pattern observed with cleavage of caspase-1. Moreover, suppression of ASC speck formation by gAcrp was restored by treatment with 3-MA, as determined by immunocytochemistry (Figure 5C). The role of autophagy induction in regulating pyroptosis was further analyzed. As indicated in Figure 5D, suppression of LDH release from primary peritoneal macrophages by gAcrp was restored significantly in the presence of 3-MA. Taken together, these data suggest that autophagy induction plays a critical role in the prevention of LPS-primed caspase-1 activation and pyroptosis by adiponectin, probably by modulating inflammasomes activation.

### 2.6. AMPK Signaling Plays A Crucial Role in Autophagy Induction and supprEssion of IL-1β Production by gAcrp in Peritoneal Macrophages

**Figure 6:**
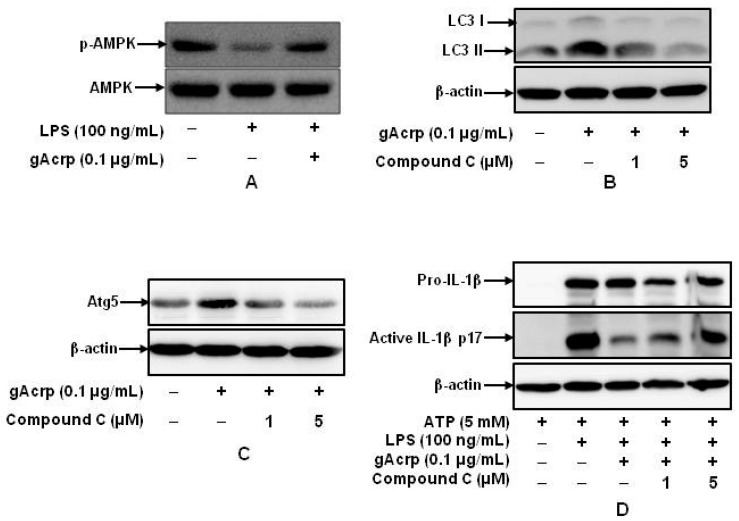
Role of 5′AMP-activated protein kinase (*AMPK*) signaling in autophagy induction and suppression of IL-1β maturation by globular adiponectin. (**A**) Peritoneal macrophages were pretreated with *gAcrp* (0.1 μg/mL) for 24 h, followed by stimulation with LPS (100 ng/mL) for additional 30 min. Phosphorylation of AMPK was determined by Western blot analysis. Images are representative of three independent experiments showing similar results; (**B**,**C**) macrophages were treated with *gAcrp* (0.1 μg/mL) for 24 h in the absence or presence of compound C. *ATG5* (**B**) and *LC3* (**C**) expression levels were assessed by Western blot analysis. Representative images from three independent experiments are shown along with β-actin as an internal loading control; (**D**) cells were pretreated with *gAcrp* (0.1 μg/mL) for 24 h in the absence or presence of compound C, stimulated with LPS (100 ng/mL) for 6 h, and then treated with ATP (5 mM) for 30 min. Levels of pro- and mature active forms of IL-1β were determined by Western blot analysis. Representative images from three independent experiments are shown along with β-actin as an internal loading control.

A growing body of evidence clearly indicates that *AMPK* signaling plays a key role in various biological responses by adiponectin. To further elucidate the molecular mechanisms underlying autophagy induction and modulation of inflammasomes activation by *gAcrp*, we examined the potential role of AMPK signaling in autophagy induction by adiponectin in macrophages. We first looked at the effects of adiponectin and LPS on *AMPK* activation. LPS treatment decreased the level of phosphorylated *AMPK*; however, as expected, pretreatment with *gAcrp* restored suppression of *AMPK* phosphorylation following LPS treatment in murine peritoneal macrophages (Figure 6A). In addition, pretreatment with compound C, a selective inhibitor of AMPK, abolished *gAcrp*-induced accumulation of *LC3* II and *ATG5* (Figure 6B,C), indicating the involvement of *AMPK* signaling in autophagy induction by *gAcrp*. The functional role of *AMPK* signaling in the suppression of IL-1β maturation by *gAcrp* was further examined. As indicated in Figure 6D, treatment with compound C significantly restored the suppressive effect of *gAcrp* on IL-1β maturation. These data collectively indicate that *AMPK* signaling plays a cardinal role in autophagy induction and suppression of LPS-induced IL-1β maturation by *gAcrp*.

### 2.7. Autophagy Induction and Suppression of The Inflammasome Activation by Globular Adiponectin Is Abrogated in AMPK Deficient Macrophages

**Figure 7:**
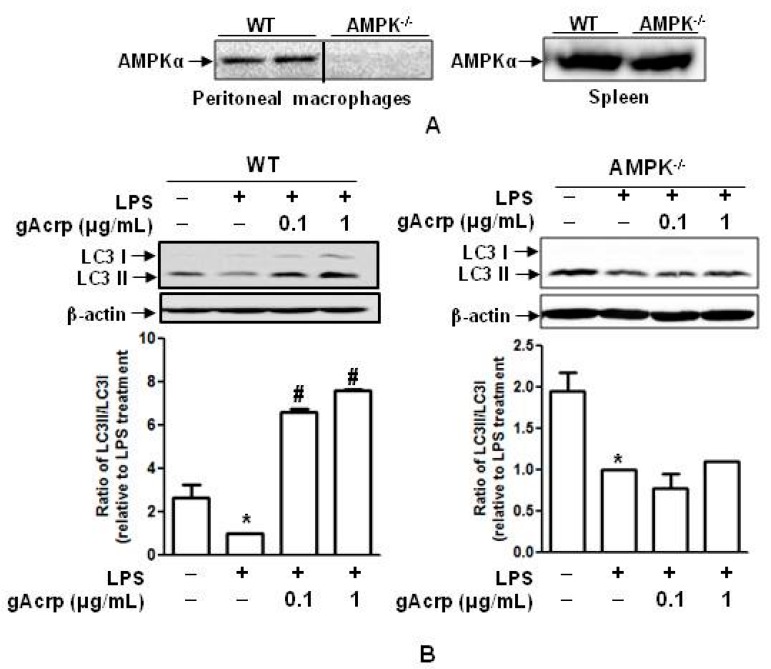

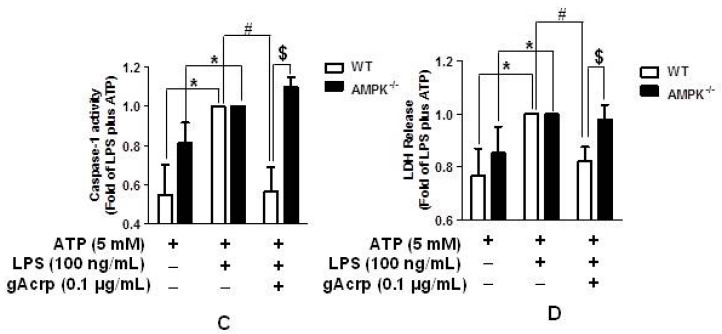
5′AMP-activated protein kinase (AMPK) signaling deficiency diminishes suppression of inflammasome activation by globular adiponectin by impairing autophagy induction. (**A**) Peritoneal macrophages and spleen cells were isolated from wild-type mice or macrophage-specific conditionally AMPK-deficient mice (AMPK^−/−^). AMPK protein expression was determined by Western blot analysis; (**B**) peritoneal macrophages were isolated from wild-type mice or macrophage-specific conditionally AMPK-deficient mice. Cells were pretreated with the indicated concentrations of gAcrp for 18 h and then stimulated with LPS (100 ng/mL) for 8 h. *LC3* protein levels were determined by Western blot analysis. Images are representative of three independent experiments showing similar results along with β-actin as an internal loading control. Quantitative analysis of *LC3II*/*LC3I* ratio was performed by densitometric analysis and shown in the lower panel. Values presented are fold change compared to LPS treatment and expressed as mean ± SEM (*n* = 3). * *p* < 0.05 compared to the control cells. # *p* < 0.05 compared to the cells treated with LPS; (**C**) Caspase-1 enzymatic activity was assessed by caspase-1 colorimetric enzyme assay as indicated in materials and methods. Values are represented as the mean ± SEM, *n* = 3. * *p* < 0.05 compared with cells treated with ATP; # *p* < 0.05 compared with the cells treated with LPS and ATP; $ *p* < 0.05 compared with wild type mice-derived macrophages treated with ATP, LPS and gAcrp; (**D**) cell culture media were prepared and used to measure LDH release. Values are represented as the mean ± SEM, *n* = 6. * *p* < 0.05 compared to cells treated with ATP; # *p* < 0.05 compared to cells treated with LPS and ATP; $ *p* < 0.05 compared to wild-type mouse-derived macrophages treated with gAcrp, LPS, and ATP.

To confirm the critical role of *AMPK* signaling in autophagy induction and suppression of the inflammasome activation by adiponectin, we generated *AMPK* knockout mice conditionally deficient in macrophage *AMPK*. As shown in Figure 7A, the *AMPK* gene was knocked out in macrophages, but not in cells isolated from the spleen (Figure 7A), confirming selective knockout of the *AMPK* gene. To further verify the functional role of *AMPK* signaling in gAcrp-induced autophagy activation, the *LC3II* expression level in response to *gAcrp* treatment was compared between AMPK^−/−^ and wild-type macrophages. As depicted in Figure 7B, whereas gAcrp treatment abrogated suppression of *LC3II* accumulation in response to *LPS* in wild-type macrophages, it failed to restore *LC3II* accumulation in AMPK^−/−^ macrophages. In addition, blockage of caspase-1 activation by *gAcrp* was not observed in AMPK^−/−^ macrophages (Figure 7C). Finally, the suppressive effect of *gAcrp* on LPS- and ATP-stimulated release of LDH, a marker of pyroptosis, was not observed in macrophages derived from AMPK^−/−^ mice (Figure 7D). Taken together, these results indicate the pivotal role of *AMPK* signaling in the prevention of the inflammasome activation and pyroptosis by gAcrp in macrophages.

## 3. Discussion

Inflammation, a complicated immune response induced by pathogenic stimuli, is commonly initiated by the production of pro-inflammatory cytokines and chemokines, which activate various immune cells and play critical roles in the immune system. Interleukin-1 (IL-1) cytokine family members act as crucial regulators in the recruitment of immune cells to sites of infection or injury. The inflammasome, composed of multi-protein components, plays a crucial role in the proteolytic activation of interleukin family cytokines through recruitment of ASC and pro-caspase-1 to NLR proteins, which leads to the catalytic cleavage of caspase-1 and consequent maturation of IL-1 β [2,7]. Although IL-1β plays an important role in normal physiological host defense processes, excessive production induces diverse pathological events. Dysregulated release of IL-1β is directly associated with the development and pathogenesis of various inflammation-mediated diseases, including arthritis, Alzheimer′s disease, allergies, and sepsis [9,35,36]. Therefore, malfunction and/or dysregulation of the inflammasome is considered one of the molecular bases of inflammatory diseases. In the present study, we observed that globular adiponectin potently suppresses LPS- and ATP-induced inflammasomes activation and IL-1β maturation in macrophages via autophagy induction and AMPK signaling-dependent mechanisms, demonstrating a novel mechanism underlying the potent anti-inflammatory effects of adiponectin.

IL-1β level is tightly regulated by two steps: transcription and activation. For the transcription (priming) step, inflammatory stimuli, such as LPS, induce transcriptional activation of IL-1β, mainly via NF-κB and the AP-1 pathway, and enhance IL-1β expression. The second signal induces maturation of IL-1β, which is mediated by inflammasomes activation. In the present study, we found that gAcrp suppressed LPS-induced pro-IL-1β and IL-1β mRNA expression, indicating that gAcrp targets and regulates transcription of IL-1β (Figure 1), which is consistent with previous reports demonstrating the inhibitory effect of gAcrp on NF-κB and AP-1. However, in these experiments, the suppressive effects on pro-IL-1β and IL-1β mRNA were lower than those observed in the generation of active IL-1β. In additional experiments to investigate the modulatory effects of gAcrp on activation (second) signal, we found that gAcrp treatment also significantly suppressed caspase-1 and the inflammasome activation (Figure 2 and Figure 3). These results clearly indicate that gAcrp regulates IL-1β at both the priming and activation signaling steps. It is well established that adiponectin regulates inflammatory responses in diverse experimental conditions via inhibition of pro-inflammatory transcription factors such as NF-κB and AP-1. However, the effects of adiponectin on the modulation of the inflammasome and their molecular mechanisms are mostly unknown. During this manuscript was being prepared, Ehsan and colleagues recently reported that adiponectin attenuated endothelial activation induced by microparticles derived from monocytes by modulating NLRP3 inflammasomes activation [37], demonstrating that pleiotropic effects of adiponectin can be mediated through the control of inflammasomes. However, the regulatory effects of adiponectin on inflammasomes in immune cells and its physiological role in the modulation of inflammatory responses are still largely unknown. In the present study, in an attempt to elucidate the molecular mechanisms underlying anti-inflammatory responses by adiponectin, we investigated the effects of globular adiponectin on the inflammasome activation in macrophages and further delineate the involvement of autophagy induction in the modulation of inflammasomes.

The inflammasome acts as a cytosolic signaling platform in the innate immune system. It is composed of multiple components, and their assembly is critical to the activation of caspase-1 and maturation of pro-inflammatory cytokines [2]. The assembly process, which involves the coupling of cytosolic sensor molecules, including NLRP1, NLRP3, and NLRC4 with caspase-1, is mediated by AS, which acts as an adaptor molecule [9]. The formation of ASC specks leads to caspase-1 activation and, consequently, maturation of IL-1β and pyroptosis. Bryan et al. reported that blockage of ASC cytosolic aggregation completely suppresses pathogen-induced inflammasomes activation [38]. In addition, deficiency of ASC by targeted gene deletion or pharmacological inhibitors repressed the activation of caspase-1 and production of mature IL-1β caused by various stimuli [34,39]. Therefore, ASC speck is considered a critical target for the regulation of inflammasomes [1,34,40]. In fact, cytokine release inhibitory drug 3 (CRID3), which blocks IL-1β production, targets ASC oligomerization during NLRP3- and AIM2 inflammasomes activation [41,42]. In the present study, we observed that treatment with gAcrp prevented LPS- and ATP-stimulated ASC speck formation in macrophages (Figure 3C) and that speck formation is modulated by autophagy activation (Figure 5C). To further investigate the underlying mechanisms, we examined the effects of gAcrp on the expression of inflammasomes components and found that gAcrp suppressed LPS-induced ASC expression (Figure 3A). In these experiments, we also observed that the basal level of ASC was high in murine peritoneal macrophages and that LPS treatment led to a slight increase in ASC expression. However, LPS-induced NLRP3 expression was not significantly affected by treatment with gAcrp (Figure 3B). These results indicate that adiponectin may turn off the signal for the inflammasome activation at the level of inflammasomes assembly, as well as modulate the expression of inflammasomes components. However, given that inflammasomes activation is a result of various priming steps, it is still possible that adiponectin regulates inflammasomes activation by modulating many other inflammasomes pathways, such as AIM2 and NLRC4, and/or other mechanisms. Further studies will be needed to gain insights into the mechanisms underlying the modulation of inflammasomes pathways by adiponectin.

Herein, we have clearly demonstrated that autophagy induction plays a key role in the prevention of inflammasomes activation by gAcrp. Biochemical regulation of the inflammasome is highly complicated, and many factors have been proposed to regulate inflammasomes function. For instance, lysosomal damage and subsequent release of cathepsin B, increased reactive oxygen species (ROS) production, and enhanced K^+^ efflux are considered typical factors that trigger activation of inflammasomes [43]. Of these, autophagy induction has been shown to efficiently suppress ROS production from damaged mitochondria via mitophagy [44]. Moreover, adiponectin exhibited potent anti-oxidant functions in various experimental conditions. Therefore, it is highly plausible that autophagy induction can contribute to suppression of the inflammasome activation by adiponectin via the suppression of ROS production. However, at this stage, the exact mechanism(s) underlying autophagy-induced suppression of the inflammasome is not well defined, and further studies seeking to understand these molecular mechanisms is worth considering.

In the present study, although we demonstrated that LPS treatment decreased *LC3II* accumulation in macrophages, and that this was restored by pretreatment with gAcrp (Figure 7B), the effects of LPS on autophagy induction are controversial. Stimulation of toll like receptors (TLRs) has been reported to trigger the induction of autophagy. For example, TLR4 stimulation by LPS induced autophagy activation via a Toll-interleukin-1 receptor domain-containing adaptor-inducing interferon-β (TRIF)-dependent, but myeloid differentiation factor 88 (MyD88)-independent, signaling pathway [45]. In addition, Delgado et al. reported that TLR ligands, including Poly(I:C) (TLR3), LPS (TLR4), and single-stranded RNA (TLR7), induced autophagy in murine bone marrow macrophages, RAW 264.7 macrophages, and J774 cells [46]. However, in the present study, we found that LPS treatment suppressed *LC3II* accumulation in murine peritoneal macrophages (Figure 7B). In line with this finding, a recent study by Saitoh and colleagues demonstrated that LPS stimulation did not increase the number of *LC3* puncta in primary macrophages [14]. Based on these controversial reports, the effects of TLR ligands, including LPS, on autophagy induction are not conclusive. These contradictory results regarding the role of TLR signaling in *LC3II* accumulation may be due to the different experimental conditions such as cell type, concentration of the stimulant used, and environments of cell growth.

5′AMP-activated protein kinase (*AMPK*) signaling has been shown to have various beneficial effects in the human body [18,47]. In addition to its critical roles in energy homeostasis and metabolic effects, *AMPK* signaling has been implicated in anti-inflammatory responses through various mechanisms [48]. In particular, increasing evidence has indicated a potential role for AMPK signaling in the regulation of the inflammasome activation. For example, enhancement of AMPK activity was shown to cause suppression of palmitate-induced inflammasomes activation [28], whereas deficiency of AMPK by a pharmacological inhibitor or siRNA enhanced IL-1β and IL-18 production in monocyte-derived macrophages from patients with type 2 diabetes [49]. Moreover, mangiferin, a bioactive xanthonoid found in plants, was shown to inhibit high glucose-induced NLRP3 inflammasomes activation through AMPK-dependent mechanisms in endothelial cells [50]. With these considerations, we hypothesized that *AMPK* signaling would play a role in the modulation of inflammasomes activation by adiponectin in macrophages. In the present study, we showed that AMPK signaling plays a critical role in the suppression of LPS-stimulated inflammasomes activation by gAcrp via modulation of autophagy induction (Figure 6 and Figure 7). In addition, we also clearly demonstrated the contribution of *AMPK* signaling to the inhibition of inflammasomes activation, IL-1β maturation/secretion, and pyroptosis by gAcrp (Figure 6) in an in vitro model of primary peritoneal macrophages. To further confirm the role of AMPK signaling, we carried out similar experiments using primary cells, in which *AMPK*α1 was conditionally knocked out in macrophages, and observed essentially similar results as those with peritoneal macrophages. Collectively, these data convincingly indicate that AMPK is a key signaling molecule that mediates the negative regulation of the inflammasome by adiponectin.

## 4. Materials and Methods

### 4.1. Materials

Recombinant human globular adiponectin (gAcrp) was obtained from Peprotech Inc. (Rocky Hill, NJ, USA). LPS, adenosine 5′-triphosphate (ATP), and 3-methyadenine (3-MA) were purchased from Sigma-Aldrich (St. Louis, MO, USA). A Membrane Permeability/Dead Cell Apoptosis Kit with PO-PRO-1 and 7-Aminoactinomycin D for Flow Cytometry were purchased from Molecular Probes (Eugene, OR, USA). A Caspase-1/ICE Colorimetric Assay Kit was obtained from Biovision (Mountain View, CA, USA), and 5-aminoimidazole-4-carboxamide-1-b-4-ribofuranoside (AICAR), a pharmacological activator of AMPK, was obtained from Calbiochem (San Diego, CA, USA). Primary antibodies against phosphorylated and total AMPKα (phospho-specific and total form), IL-1β, and *LC3* were obtained from Cell Signaling Technology Inc. (Beverly, MA, USA), β-actin was obtained from Thermo Scientific (Hudson, NH, USA), and NLRP3 was purchased from R&D Systems (Minneapolis, MN, USA). Antibodies against caspase-1 and ASC were purchased from Adipogen (San Diego, CA, USA). Secondary goat-anti-rabbit and anti-mouse antibodies were obtained from Thermo Scientific.

### 4.2. Generation of Macrophage-Specific AMPK Knockout Mice

Mice with a loxP-flanked AMPK allele (AMPK*^flox/flox^*) on a C57BL/6 background were purchased from The Jackson Laboratory (Bar Harbor, ME, USA). Lyz2-Cre mice (Jackson Laboratory) have a nuclear-localized Cre recombinase inserted into the first coding *ATG* of the lysozyme 2 gene (*Lyz2*). To generate macrophage-specific AMPK conditional knockout mice, AMPK-deficient mice (AMPK*^flox/flox^*) were crossed with *Lyz2*-Cre transgenic mice, which express macrophage-specific Cre recombinase, resulting in the production of age-matched *AMPK*^+/+^*Lyz2*^Cre/Cre^ (wild-type, WT) and *AMPK^flox/flox^Lyz2*^Cre/Cre^ (macrophages-specific AMPK knockout, AMPK^−/−^). AMPK knockout and WT mice were maintained in the specific pathogen-free facility of the Yeungnam University Animal Research Center. CO_2_ inhalation using gradual fill method was used for euthanasia to minimize potential pain. All the animal experiments were conducted in accordance with protocols reviewed(protocol # 2016-02-01) and approved by the Institutional Animal Care and Use Committee of the Yeungnam University Animal Research Center (approval code 2016-009).

### 4.3. Isolation and Culture of Murine Peritoneal Macrophages

Murine peritoneal macrophages were isolated as described previously [18]. Briefly, 8-to-12-week-old female C57BL/6 mice were intraperitoneally (I.P.) injected with 1 mL of 4% Brewer thioglycollate medium. Two days after injection, cells in the peritoneum were washed with ice-cold Hank’s balanced salt solution (HBSS, calcium- and magnesium-free) three times. Media prepared from peritonea were centrifuged at 12,000 rpm for 5 min, and pellets were resuspended in RBC lysis buffer (BioLegend, San Diego, CA, USA). After washing with HBSS, the pellets were resuspended in RPMI 1640 media supplemented with 10% fetal calf serum (FCS) and 1% (*v/v*) penicillin-streptomycin. Cells were then seeded on culture plates and routinely cultured at 37 °C in an incubator with a humidified atmosphere of 95% of oxygen and 5% CO_2_.

### 4.4. Preparation of Cellular Extracts and Western Blot Analysis

Peritoneal macrophages were seeded in 35-mm dishes at a density of 1 × 10^6^ cells. After overnight culture, cells were treated with gAcrp, LPS, and ATP as indicated, and then total proteins were extracted using RIPA lysis buffer containing Halt Protease and Phosphatase Inhibitor Cocktail (Pierce, Rockford, IL, USA). To detect the levels of secreted IL-1β, cell culture media were collected and the proteins in the media were precipitated using trichloroacetic acid (TCA) protein precipitation method. The culture media (800 µL) were incubated with TCA (200 µL) for 10 min at 4 °C. After centrifugation at 13,200 rpm for 15 min, the pellets were collected, washed with cold acetone two times and dried by placing tube in 94 °C heat block. The precipitates were then dissolved with the sample buffer. For the immunoblot analysis, 20–30 μg of protein was loaded onto a 10–15% sodium dodecyl sulfate-polyacrylamide gel electrophoresis (SDS-PAGE) gel, transferred to polyvinylidene fluoride (PVDF) membranes, and blocked with 5% skim milk in phosphate-buffered saline (PBS)/Tween 20 for 1 h. Membranes were incubated with the designated primary antibody overnight at 4 °C. After washing with PBS/Tween three times, membranes were incubated with secondary antibody conjugated with horseradish peroxidase (HRP) for 1 h. Images of the blots were detected by chemiluminescence using Pierce ECL Western blotting substrate and were captured with a Fujifilm LAS-4000 Mini (Fujifilm, Tokyo, Japan).

### 4.5. Immunocytochemistry

For detection of ASC speck formation, cells were seeded in 8-well chamber slides at a density of 2 × 10^4^ cells/well and treated with gAcrp and/or LPS. Cells were washed twice with PBS, fixed with 4% paraformaldehyde solution for 20 min, permeabilized with 0.2% Triton X-100 for 10 min, and then blocked with 3% bovine serum albumin in 1× PBS for 1 hat room temperature before incubating with anti-ASC (1:100) overnight at 4 °C. After washing, cells were incubated with FITC-conjugated goat anti-rabbit secondary antibody (Pierce) for 1 hat room temperature, rinsed with 1× PBS, and incubated for 10 min with 4′,6-diamidino-2-phenylindole (DAPI, 1:1000). Images were captured by fluorescence microscopy (Nikon, Tokyo, Japan).

### 4.6. Enzyme-Linked Immunosorbent Assay (ELISA)

Macrophages were seeded in 96-well plates at a density of 1 × 10^5^ cells per well. After incubation overnight, cells were pretreated with 0.1 μg/mL of gAcrp for 18 h, followed by stimulation with LPS (100 ng/mL) for 24 h and ATP for an additional 30 min. The supernatants were then collected and used for measurement of IL-1β using ELISA kits (BD Biosciences, San Diego, CA, USA) according to the manufacturer’s instructions.

### 4.7. Caspase-1 Enzyme Activity Assay

Caspase enzyme activity was determined using a caspase-1 enzymatic assay kit (Biovision) according to the manufacturer’s instructions. Murine peritoneal macrophages were seeded in 60-mm dishes at a density of 3 × 10^6^ cells per dish. After overnight incubation, cells were pretreated with gAcrp for 18 h, stimulated with LPS for 6 h, and then treated with 5 mM ATP for 30 min. Cell lysates were prepared and used for the measurement of spectrophotometric chromophore p-nitroanilide (pNA) after cleavage from the labeled substrate YVAD-pNA using a microplate reader (SPECTROstar Nano, BMG Labtech, Ortenberg, Germany).

### 4.8. Measurement of Lactate Dehydrogenase (LDH) Release

The release of LDH was measured using CytoTox 96 Non-Radioactive Cytotoxicity Assay (Promega, Medison, WI, USA) according to the manufacturer’s instructions. Briefly, macrophages were seeded in 96-well plates at a density of 1 × 10^5^ cells per well. After incubation overnight, cells were pretreated with gAcrp for 18 h, followed by stimulation with LPS and ATP as indicated in the figure legends. The culture media were collected and assayed for LDH activity based on the conversion of tetrazolium salt into a red formazan product. Activity was determined by measuring the absorbance at 490 nm using a SPECTROstar Nano (BMG Labtech).

### 4.9. RNA Isolation, Reverse Transcription (RT), and Quantitative Real-Time PCR (qPCR)

To measure IL-1β mRNA levels, total RNA was isolated using Qiagen lysis solution (Qiagen, Frederick, MD, USA) according to the manufacturer’s instruction. Total RNA was reverse transcribed into cDNA, and quantitative real time PCR (qPCR) was then performed using ABsolute qPCR SYBR Green Capillary Mix (Thermo Scientific, Renfrew, UK) at 95 °C for 15 min, followed by 40 cycles at 95 °C for 15 s, 56 °C for 30 s, and 72 °C for 45 s. The amount of target mRNA was analyzed using the comparative threshold (*C*_t_) method after normalizing the target mRNA *C*_t_ values to those of glyceraldehyde-3-phosphate dehydrogenase (GAPDH) (Δ*C*_t_). The primer sequences used for amplification were as follows. Forward primer; 5′-GCCTCGTGCTGTCGGACCCATAT-3′, Reverse primer 5′-TCCTTTGAGGCCCAAGGCCACA-3′.

### 4.10. Statistical Analysis

Values were presented as mean ± standard error of the mean (SEM) derived from at least three separate experiments. Data were assessed by one-way analysis of variance (ANOVA) and Tukey’s multiple comparison tests using GraphPad Prism version 5.01 (La jolla, CA, USA). Differences between groups were considered to be significant at *p* < 0.05.

## 5. Conclusions

**Figure 8:**
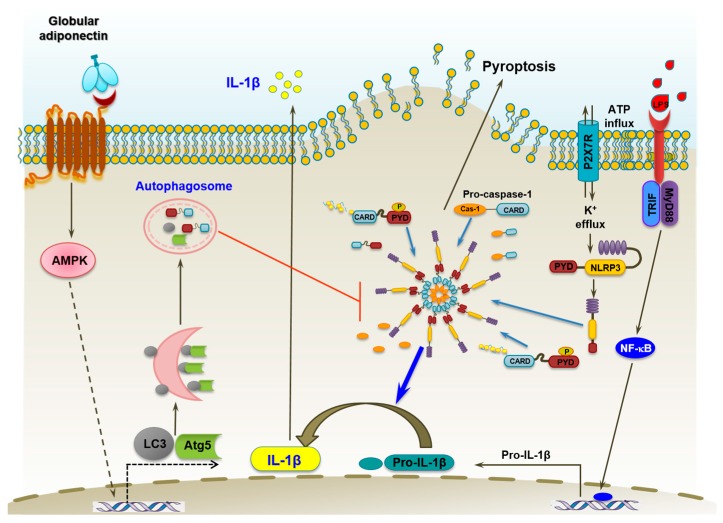
Proposed model for the suppression of lipopolysaccharide (LPS)-primed inflammasomes activation by globular adiponectin via autophagy induction in macrophages. Treatment with globular adiponectin induces activation of an autophagic process via 5′AMP-activated protein kinase (*AMPK*) signaling in murine macrophages. The detailed mechanisms underlying p62 induction by globular adiponectin in the context of AMPK signaling remain to be determined. LPS treatment has been shown to enhance expression of pro-IL-1β through TLR4 and NF-κB signaling. Extracellular ATP triggers the inflammasome activation through a series of biological processes leading to the cleavage and activation of pro-caspase-1, which further induces maturation and secretion of IL-1β and pyroptosis in immune cells. In this study, we clearly showed that globular adiponectin suppresses LPS-stimulated IL-1β maturation and pyroptosis via modulation of inflammasomes activation. Importantly, autophagy induction plays a crucial role in the modulation of inflammasomes activation by adiponectin. It is likely that adiponectin-induced autophagy activation regulates inflammasomes activation through inhibition of ASC speck formation and inflammasomes assembly. Furthermore, *AMPK* signaling plays a pivotal role in autophagy activation and prevention of inflammasomes activation by globular adiponectin. P2X7 receptor: Purinergic ATP receptor; PYD: PYRIN-PAAD-DAPIN domain; CARD: Caspase activation and recruitment domain.

In conclusion, the data presented in this study have demonstrated for the first time that globular adiponectin prevents LPS-primed inflammasomes activation and suppresses production of active IL-1β and pyroptosis in macrophages through modulation of autophagy and AMPK signaling (Figure 8). These findings not only elucidate the physiological role of adiponectin in the modulation of inflammasomes, but also provide novel insight into the molecular mechanisms underlying the relationship between autophagy and inflammasomes activation. Modulation of the inflammasome is a novel area of innate immune research related with adiponectin. In consideration of the critical role of inflammasomes and IL-1β in the pathogenesis of various diseases associated with inflammation, we suggest that regulation of inflammasomes is a mechanism involved in the anti-inflammatory responses by adiponectin.

## Supplementary Materials

Supplementary materials can be found at www.mdpi.com/1422-0067/18/6/1275/s1.
