# Supplementary material for: Globular Adiponectin Inhibits Lipopolysaccharide-Primed Inflammasomes Activation in Macrophages via Autophagy Induction: The Critical Role of AMPK Signaling
Source: Int J Mol Sci. 2017 Jun 15;18(6):1275. doi: 10.3390/ijms18061275 (PMC5486097; doi:10.3390/ijms18061275)
Supplement: Supplementary file 1 [file ijms-18-01275-s001.pdf]

Supplementary Materials:

Globular Adiponectin Inhibits Lipopolysaccharide-primed Inflammasomes Activation in Macrophages via Autophagy Induction: The Critical Role of AMPK Signaling

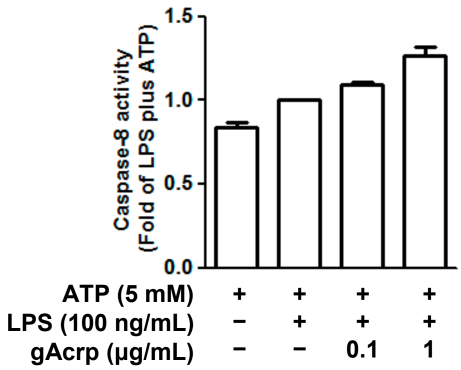

Figure S1. Effect of globular adiponectin on caspase-8 activity in primary murine peritoneal macrophages stimulated with LPS. Cells were treated with the indicated concentration of gAcrp for 18 hours followed by stimulation with LPS (100 ng/mL) for additional 6 hours prior to ATP (5 mM) treatment for 30 min. Caspase-8 enzymatic activity was determined as indicated in materials and methods. Data are expressed as fold change relative to LPS and ATP treatment. Values are shown as the mean ± SEM, $n = 3$. * $p < 0.05$ compared with cells treated with ATP.

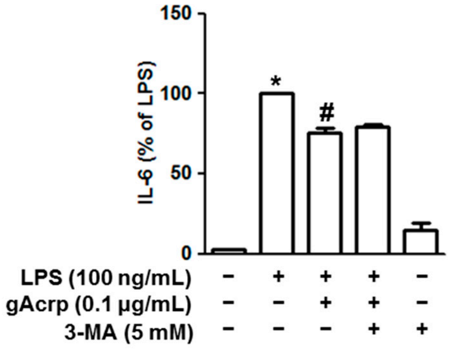

Figure S2. Role of autophagy induction in the suppression of LPS-induced IL-6 production by gAcrp. Cells were pretreated with 3-MA (5 mM) for 1 h followed by incubation with gAcrp (0.1 µg/mL) for additional 18 h. Cells were then treated with LPS (100 ng/mL) for 24 h. The cell culture media were collected and used for the measurement of IL-6 secretion by ELISA. Data are expressed as relative to LPS treatment. Values are shown as the mean ± SEM, $n = 3$. * $p < 0.05$ compared with control: # $p < 0.05$ compared with cells treated with LPS.
